# Complications of Antiretroviral Therapy Initiation in Hospitalised Patients with HIV-Associated Tuberculosis

**DOI:** 10.1371/journal.pone.0054145

**Published:** 2013-02-08

**Authors:** Helen van der Plas, Graeme Meintjes, Charlotte Schutz, Rene Goliath, Landon Myer, Dorothea Baatjie, Robert J. Wilkinson, Gary Maartens, Marc Mendelson

**Affiliations:** 1 Division of Infectious Diseases and HIV Medicine, Department of Medicine, University of Cape Town, Cape Town, South Africa; 2 Clinical Infectious Diseases Research Initiative, Institute of Infectious Diseases and Molecular Medicine, University of Cape Town, Cape Town, South Africa; 3 Department of Medicine, Imperial College London, London, United Kingdom; 4 Centre for Infectious Diseases Epidemiologic Research, School of Public Health & Family Medicine, University of Cape Town, Cape Town, South Africa; 5 Brooklyn Chest Hospital, Cape Town, South Africa; 6 MRC National Institute for Medical Research, London, United Kingdom; Indiana University and Moi University, United States of America

## Abstract

**Background:**

HIV-associated tuberculosis is a common coinfection in Sub-Saharan Africa, which causes high morbidity and mortality. A sub-set of HIV-associated tuberculosis patients require prolonged hospital admission, during which antiretroviral therapy initiation may be required. The aim of this study was to document the causes of clinical deterioration of hospitalised patients with HIV-associated tuberculosis starting antiretroviral therapy in order to inform healthcare practice in low- to middle-income countries.

**Methods:**

Prospective, observational cohort study of adult inpatients with HIV-associated tuberculosis starting antiretroviral therapy in a dedicated tuberculosis hospital in Cape Town, South Africa. Causes of clinical deterioration and outcome were recorded in the first 12 weeks of antiretroviral therapy. Patients with rifampicin-resistant tuberculosis were excluded.

**Results:**

Between May 2009 and November 2010, 112 patients (60% female), with a median age of 32 years were enrolled. At baseline the median CD4 count was 55 cells/mm^3^ (IQR 31–106) and HIV viral load 5.6 log copies/mL. All patients had significant comorbidity: 82% were bed-bound, 65% had disseminated tuberculosis and 27% had central nervous system tuberculosis. Seventy six patients (68%) developed 144 clinical events after starting antiretroviral therapy. TB-IRIS, hospital-acquired infections and significant drug toxicities occurred in 42%, 20.5% and 15% of patients respectively. A new opportunistic disease occurred in 15% of patients and a thromboembolic event in 8%. Mortality during the 12 week period was 10.6%.

**Conclusions:**

High rates of TB-IRIS, hospital-acquired infections and drug toxicities complicate the course of patients with HIV-associated tuberculosis starting antiretroviral therapy in hospital. Despite the high morbidity, mortality was relatively low. Careful clinical management and adequate resources are needed in hospitalised HIV-TB patients in the 1^st^ three months following ART initiation.

## Introduction

The World Health Organisation estimates that over 80% of all cases of HIV-associated tuberculosis (HIV-TB) occur in Africa. [1] HIV-TB causes significant morbidity with high case fatality rates during treatment (18.8%, 95% CI: 14.8–22.8%) [2], especially in patients with advanced immunodeficiency. Many of the deaths occur early after tuberculosis diagnosis. [3] Concomitant antiretroviral therapy (ART) reduces mortality in patients with HIV-TB [3] and, in patients with very advanced immunosuppression (CD4<50 cells/mm^3^), outcomes are best if ART is started ∼2 weeks after commencing tuberculosis treatment.[4]–[6] Paradoxical tuberculosis-immune reconstitution inflammatory syndrome (TB-IRIS) and significant drug toxicity are common complications in patients who start ART soon after tuberculosis treatment.[4]–[6] Other potential causes for clinical deterioration in this group include complications due to tuberculosis (e.g. haemoptysis, pneumothorax), new opportunistic diseases, drug resistant tuberculosis, bacterial infections, venous thromboembolism and drug-drug interactions.[7]–[9].

HIV-TB patients with severe immunocompromise commonly have disseminated tuberculosis and significant comorbidity, and often require admission to longterm care facilities or dedicated tuberculosis hospitals. In Sub-Saharan Africa the majority of ART programs are outpatient-based requiring regular clinic attendance prior to starting ART. Few patients start ART in hospital, and studies that report the complications and outcomes among inpatients starting ART are limited. We aimed to describe the causes for clinical deterioration in hospitalised HIV-TB patients starting ART in order to inform resource allocation of diagnostic and therapeutic services required at tuberculosis hospitals in low- and middle-income countries.

## Methods

### Design and Setting

A prospective, observational cohort study of hospitalised patients with HIV-TB was conducted at Brooklyn Chest Hospital (BCH) from 29 April 2009 until 28 February 2011. BCH is a district level tuberculosis hospital serving the Cape Town metropole. Inpatients at BCH are either too ill for ambulatory care or have drug-resistant TB, and are transferred in from hospitals or TB clinics with a diagnosis of tuberculosis already made and TB treatment initiated. The adult inpatient population has been previously described; where more than two-thirds of patients in the drug-sensitive wards are HIV-co-infected, of whom 98% had significant comorbidities and 60% had a Karnofsky performance score of ≤30. [10] An integrated ART program was started at BCH in 2008.

### Study Population and Eligibility

Eligible patients were adults (≥18 years), hospitalised at BCH with confirmed HIV infection and a diagnosis of pulmonary (PTB) or extrapulmonary tuberculosis (EPTB), not on ART, who fulfilled national criteria for starting ART at the time of the study; CD4 count <200 cells/mm^3^ or WHO Stage 4 defining illness until 31 March 2010, following which guidelines changed to include all patients with HIV-TB and CD4 count ≤350 cells/mm^3^. Exclusion criteria included patients with known rifampicin-resistant tuberculosis and those unable to provide informed consent.

First episodes of tuberculosis were treated as per national policy with standard fixed dose combination tablets composed of rifampicin (R), isoniazid (H), pyrazinamide (Z), and ethambutol (E) for the 2-month intensive phase followed by RH for the 4-month continuation phase. For retreatment tuberculosis, RHZE was given for 3 months with 40-doses of streptomycin followed by RHE for 5 months. Prior to 1^st^ April 2010, first-line ART comprised stavudine (d4T), lamivudine (3TC) and efavirenz as the preferred non-nucleoside reverse transcriptase inhibitor (NNRTI) in patients on concurrent TB therapy. In April 2010 new national guidelines were introduced with tenofovir (TdF) replacing d4T. Unless contraindicated, all patients received cotrimoxazole 960 mg daily as prophylaxis, and pyridoxine 25–50 mg daily during tuberculosis treatment as well as subcutaneous unfractionated heparin (5000 IU subcutaneously 12 hrly) for deep vein thrombosis prophylaxis if bedbound.

### Study Procedure

Follow-up was for 12 weeks after starting ART. Patients were assessed at 8 time-points: enrolment, ART initiation, then weekly for four weeks and again at week 8 and week 12 after ART initiation. At each scheduled visit, an infectious diseases specialist examined patients and targeted laboratory (including bacterial cultures and serum cryptococcal antigen) and radiological investigations were performed if there was clinical deterioration. Baseline clinical and laboratory data was recorded: age, gender, CD4 T-lymphocyte count (CD4 count), HIV viral load, WHO stage, comorbid condition(s), weight and ambulatory status. Reasons for not being on ART at admission and prior ART exposure was established. Tuberculosis data included: prior tuberculosis episode(s), site of tuberculosis (EPTB/PTB), diagnostic method (smear, culture, clinical), and TB drug details (susceptibility, regimen, duration, serious adverse drug reactions prior to starting ART). Unscheduled visits and additional investigations occurred in the event of clinical deterioration. Routine care was the responsibility of the ward doctor and nursing staff.

We recorded the following outcome measures in the event of a clinical deterioration: new WHO stage 4 defining condition, drug-induced toxicity requiring a drug switch, hospital acquired infection, TB-IRIS, and significant other comorbidity necessitating investigation and treatment.

### Definitions

Tuberculosis was diagnosed on the basis of smear or culture positivity. Where this was negative or unavailable, diagnosis was in line with WHO guidelines for diagnosis of smear negative or extrapulmonary tuberculosis in HIV-1 infected persons. [11] TB-IRIS was diagnosed according to the International Network for the Study of HIV-associated IRIS (INSHI) case definitions. [12] Hospital acquired infection (HAI) was defined as an infection acquired after 48 hours of hospital admission that was not clinically apparent at the time of the patient’s hospital admission. When organisms were cultured in the absence of an inflammatory response, the isolate was regarded as a colonising microorganism, and not classified as a HAI. [13].

### Ethics

The study was approved by the University of Cape Town’s Faculty of Health Sciences Research Ethics Committee (HREC No: 049/2009) and the Provincial Research Committee of the Department of Health, Western Cape. All participants signed written informed consent.

### Statistical Analysis

Data were analysed using Stata version 12.0 (Stata Corporation, USA). Measures were described with medians and interquartile ranges (IQR) or proportions. We used chi-square tests (replaced by exact tests for sparse data) to compare proportions, rank-sum tests to compare medians, and t-tests to compare mean values between groups. Kaplan-Meier methods were used to describe the survival of patients over time.

## Results

### Patient Characteristics

**Figure 1:**
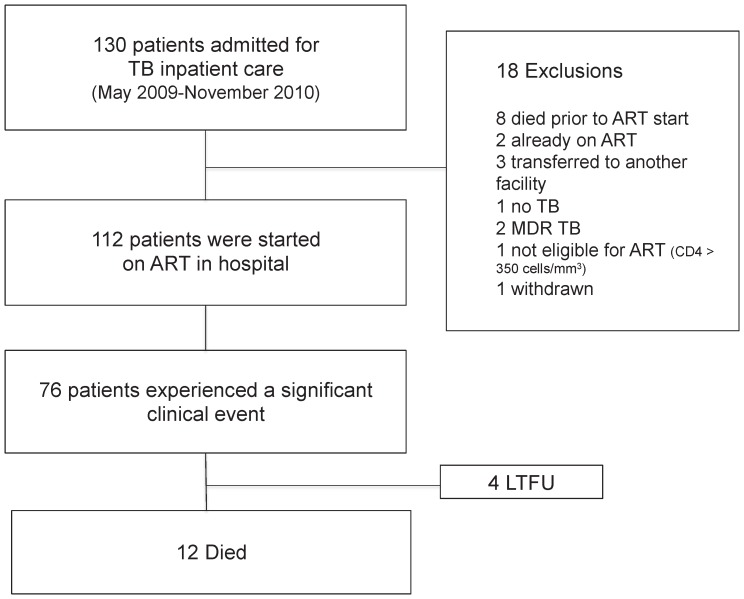
Patient enrollment. LTFU = lost to follow-up, TFO = transferred out.

A total of 130 participants were screened for enrollment (figure 1), 112 of whom met inclusion criteria and started ART in hospital. Baseline characteristics are shown in table 1. The cohort was characterised by advanced immunosuppression with WHO stage 4 disease in the majority, very low median CD4 cell count, high baseline HIV viral load and 81% were bed-bound. Approximately two thirds had disseminated tuberculosis and only a minority, 17% and 18% respectively, had exclusively pulmonary or extrapulmonary TB at a single site. Just over half of patients were presenting with a repeat episode of tuberculosis and 50% were newly diagnosed with HIV.

**Table 1:** Baseline Characteristics of 112 HIV-TB Inpatients starting ART.

Age: years, median (IQR)	32 (27–40)
Female gender, n (%)	67 (60)
CD4 count, cells/mm^3^, median, (IQR)	55 (31–106)
HIV viral load, log copies/mL, median (IQR)	5.6 (5.1–6.1)
WHO Stage 4, n (%)	97 (87)
Haemoglobin, g/dL, median (IQR)	9.0 (8.7– 10.3)
Weight, kg, median (IQR)	46 (39–52)
Bed bound: n (%)	91 (82)
Corticosteroids at baseline: n (%)	25 (22)
**Tuberculosis**	
Diagnosis microbiologically confirmed: n (%)*	86 (77)
Previous TB: n (%)	66 (59)
Exclusively PTB: n (%)	19 (17)
EPTB at single site: n (%)	20 (18)
Disseminated TB: n (%)	73 (65)
Neurological TB: n (%)	30 (27)

* 82 cultured MTB, and 4 only smear positive.

The median time from the start of anti-tuberculosis treatment to ART start was 36 days (IQR, 27–57) and the majority (95%) received an efavirenz-based regimen, more commonly with tenofovir than stavudine (Table 2). Twenty-four patients (21%) were receiving corticosteroids at the time of starting ART; the main indication for using corticosteroids was for TB meningitis in 20 patients. Ninety-six patients completed 12-weeks of follow-up, with 4 patients absconding from hospital after completing 8 weeks of inpatient ART.

**Table 2:** Antiretroviral therapy and duration of hospitalization.

Reason not on ART at enrollment	
New HIV diagnosis: n (%)	57 (51)
Did not fulfill criteria for ART previously §, n (%)	13 (12)
Personal reasons e.g. denial, n (%)	11 (10)
**ART**	
ART naïve, n (%)	109 (97)
Median time from starting TB treatment to ART start,days (IQR)	36 (27–57)
Median time from hospitalization to ART start,days (IQR)	16(12–23)
**ART regimen, n (%)**	
D4T 3TC EFV	43 (38)
TdF 3TC EFV	54 (48)
AZT 3TC EFV	10 (9)
D4T 3TC NVP	4 (4)
AZT, 3TC, lopinavir/ritonavir	1 (1)
**Hospital stay**	
Duration of admission at referral hospital, days,median (IQR)	15 (11–31)
Length of admission at BCH*	99 (75–130)

§ CD4>200 cells/mm^3^, WHO clinical stage 1–3.

* Information available for 94 subjects only, deaths excluded.

### Clinical Deterioration

Overall 76 (68%) patients experienced a significant clinical deterioration after initiating ART. A total number of 144 events were recorded with a median of 2 events (range 1–5) per patient. Paradoxical TB-IRIS, HAI, drug toxicity (most commonly efavirenz-related neuropsychiatric toxicity) and unmasking of opportunistic diseases (most commonly oesophageal candidiasis, *Pneumocystis jirovecii* pneumonia and Kaposi’s sarcoma) were the main reasons for deterioration (Table 3).

**Table 3:** Causes of clinical deterioration.

Cause	n (%)
Tuberculosis – IRIS	47 (42)
Drug Toxicity	23 (20.5)
Hospital acquired infection	23 (20.5)
Opportunistic disease (includes Kaposi’s sarcoma)	17 (15)
Deep vein thrombosis or pulmonary embolism	9 (8)
Herpes virus reactivation	8 (7)
Other	17 (15)

* Patients commonly had more that one episode of clinical deterioration, in total 144 episodes of deterioration were recorded.

n = 144*.

Paradoxical TB-IRIS occurred in 42% of patients. The median duration from starting ART to the onset of TB-IRIS symptoms was 12 days (range 4–49 days). Seven patients developed potentially life-threatening IRIS, 6 due to worsening tuberculous meningitis and one due cardiac tamponade requiring urgent pericardiocentesis. The only baseline variable that was significantly associated with the development of TB-IRIS on univariate analysis was CRP>10 mg/L (Hazard ratio; 95% confidence interval: 1.7 [1.1–2.6]). Corticosteroid use at the time of ART initiation was associated with a 40% reduction in TB–IRIS incidence, however this was not statistically significant (p = 0.06).

Twenty-three patients (20.5%) developed an HAI, the commonest being urinary tract sepsis in 9 patients. Seven patients had sepsis syndrome with a bloodstream infection, 3 pneumonia, 3 *Clostridium difficile* diarrhoea and one patient developed chickenpox acquired following an exposure to another patient with herpes zoster. Thirteen isolates were identified in the 22 patients with bacterial sepsis, 9 of which were multi-drug resistant bacteria: 8 extended spectrum β-lactamase (ESBL)-producing enterobacteriaceae (7 from urine culture and 1 blood culture) and 1 methicillin-resistant *Staphylococcus aureus* (sputum culture).

### Mortality

**Figure 2:**
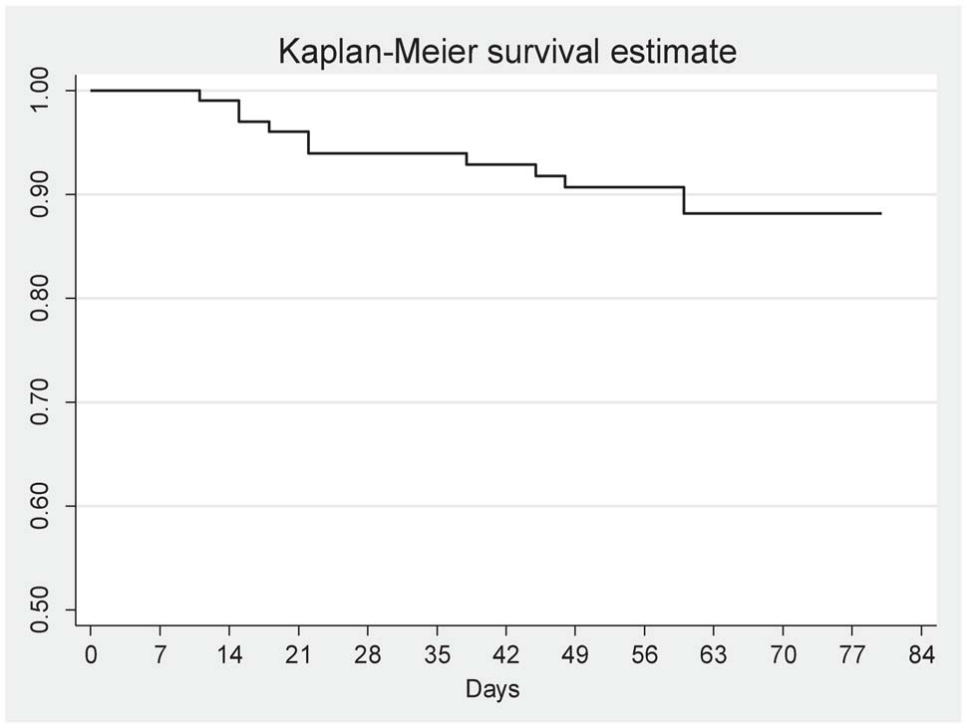
Kaplan-Meier curve showing survival of 112 patients with HIV-TB, from the start of antiretroviral therapy.

Twelve patients died during the 12-week follow-up period resulting in cumulative mortality of 10.6% (Figure 2). Neurological tuberculosis, weight <50 kg, corticosteroid use at time of ART initiation, being bed-bound, or CD4 cell count <50 cells/mm^3^ were not significantly associated with mortality. The cause of death was ascribed to sepsis in 11 patients and pulmonary embolism in one. None of the deaths were thought to be directly caused by paradoxical TB-IRIS.

## Discussion

Our study demonstrated that over two-thirds of hospitalised HIV-TB patients develop complications resulting in clinical deterioration after starting ART as inpatients. Over 40% experienced paradoxical TB-IRIS, with HAIs, drug toxicities, and opportunistic diseases being other frequent causes of deterioration. A novel finding is that HAIs were common (occurred in 20.5%), and 11 of the 12 deaths were attributed to sepsis. Reassuringly, despite the high incidence of clinical deterioration, 89% of participants were alive three months after ART initiation. Adequate clinical care and diagnostic and therapeutic resources are required to manage this HIV- as well as tuberculosis-associated morbidity.

HIV infection, especially with a low CD4 count, is a risk factor for clinical deterioration and death on TB treatment. [9] A previous study conducted in Cape Town of 292 ambulatory TB patients, 209 of whom were HIV-infected, demonstrated that 40% deteriorated while on TB therapy, 26% required hospitalization, and 15% died during the 6 month follow-up period. [9] Training health care workers on the causes, recognition and management of deterioration in HIV-TB patients, in addition to training on ART management, is required to optimize the outcome of HIV-TB patients.

The high incidence of paradoxical TB-IRIS in our study (42%) corresponds with the higher estimates reported from previous cohort studies (8–43%) [14]. Our patients had many of the risk factors for developing TB-IRIS; low CD4 counts, disseminated tuberculosis and relatively short time interval between starting tuberculosis therapy and ART. Our findings add to the evidence that paradoxical TB-IRIS is an uncommon cause of mortality [15], even in very ill HIV-TB patients starting ART in hospital. One caveat is that 7 of 47 IRIS cases developed potentially life-threatening manifestations and these were promptly ascertained and managed by specialists with early corticosteroid introduction or dose escalation. The association of a raised CRP prior to starting ART with paradoxical TB-IRIS has been previously described. [16] Being on corticosteroids at the time of initiating ART showed a trend towards reduced risk for TB-IRIS, although this was not statistically significant in this small study. Larger prospective studies would be necessary to evaluate this properly.

The large number of HAIs we diagnosed, and the associated mortality is alarming. Our findings suggest that once patients with advanced HIV and TB are established on appropriate TB treatment, ART and cotrimoxazole, then bacterial infections emerge as the most important cause of death. We suspect that colonization with multi-drug resistant bacteria occurred in the referring general hospital, as antibiotic selection pressure in TB hospitals is likely to be low. High rates of urinary catheterization during the acute admission at the referring hospital, is a likely contributing risk factor to the urinary tract infections. Depressed monocyte responses may be one of the reasons why HIV-infected patients with advanced immunosuppression are at high risk of bacterial infections. [17] Our study and a previous study from a hospitalized cohort in Cape Town [8], confirm that drug-resistant bacteria which require the use of carbapenems and other costly antibiotics are causing HAI. These antibiotics are generally unavailable at district or secondary level hospitals in developing countries and there is therefore often a delay in treating these infections appropriately resulting in high mortality. Furthermore, because the risk of multi-drug resistance is high in HAIs, it is essential to culture clinical specimens prior to commencement of antibiotics. Our findings emphasize not only the need for appropriate antibiotics to be available, but for basic infection prevention control practices, most importantly effective hand disinfection, to be re-inforced and practiced to prevent secondary spread of infection. Reducing the number of days that patients have urinary catheters in situ, and wherever possible avoiding the need for indwelling intravenous catheters are also important interventions. Every attempt is made to limit duration of hospital stay. However, severity of illness and extremely poor social circumstances often preclude early discharge.

Our study has several limitations. The follow-up period was limited to the first 3 months of ART, so events occurring in the latter half of TB therapy were not ascertained. The generalizability of our findings is limited by the fact that all participants in the study were evaluated by a specialist physician trained in infectious diseases at study visits, which could in part explain our relatively low mortality. There was no control group to ascertain outcome of patients who did not receive input from an Infectious Diseases specialist. Another factor limiting generalizability of our outcomes is that BCH is relatively well-resourced with access to radiology and laboratory services. It is unlikely that such good outcomes can be achieved in less well-resourced facilities, which would be found in the majority of TB hospitals in low- to middle-income countries. However, specialist input and access to diagnostic facilities allowed us to ascertain the frequency and type of complications occurring in sick HIV-TB inpatients starting ART. Another limitation is that not all patients developing sepsis had appropriate cultures sent, as transportation of specimens to the off-site microbiology laboratory occurs only once a day during weekdays. In addition the causes of death were ascertained by the attending infectious diseases specialist and no post-mortem studies were performed on the patients. For the analysis of predictors of mortality the sample size was to too small to allow for meaningful comparison between groups.

Our findings have important implications for service delivery and resource allocation at hospitals offering dedicated tuberculosis services. HIV-TB patients requiring long term admission are a vulnerable group with advanced immunosuppression, frequently have disseminated tuberculosis, and are at high risk of early mortality if ART is not initiated timeously. Three landmark trials have shown that ART initiated within 2–3 weeks after starting TB therapy in patients with HIV-associated tuberculosis and a CD4<50 cells/mm^3^ results in a reduction in death and AIDS.[4]–[6] Where HIV-TB services are not integrated, hospitalized HIV-TB patients are referred to primary community ART clinics after discharge, resulting in unnecessary delay of ART initiation and this likely increases mortality. If HIV-TB patients have to attend outpatient ART clinics to initiate ART during their hospital stay, there is a risk of transmitting *M. tuberculosis*, including drug-resistant strains to the HIV-infected clinic population. Hence, integrating ART initiation into tuberculosis hospitals is vital to expedite ART initiation as well as to prevent TB transmission in ART clinics. With proper human resource allocation to tuberculosis hospitals such as BCH, ‘fast-track’ counselling can be employed to ensure patients are treatment ready. Patients in our study started ART a median of 36 days after TB treatment, much of the delay occurring because of the period it took for patients to be admitted to BCH after TB diagnosis.

The strength of our study is that it illustrates the clinical complexity of starting ART in hospitalized HIV-TB inpatients and the need for clinicians trained in the clinical management of these very ill patients. It argues strongly for proper training and resource allocation to manage this vulnerable group of patients.

### Conclusions

High rates of paradoxical TB-IRIS, HAIs, drug toxicities and new opportunistic infections occur in hospitalised HIV-TB inpatients with advanced immunosuppression initiating ART. Despite the high morbidity, relatively good short-term outcomes can be achieved with careful clinical management. There is a need for prospective studies in other settings, and for additional studies evaluating the effect of training and the provision of ART to HIV-TB inpatients.
